# Postharvest dormancy-related changes of endogenous hormones in relation to different dormancy-breaking methods of potato (*Solanum tuberosum* L.) tubers

**DOI:** 10.3389/fpls.2022.945256

**Published:** 2022-08-10

**Authors:** Muhammad Wasim Haider, Muhammad Nafees, Ishtiaq Ahmad, Baber Ali, Maryam, Rashid Iqbal, Dan C. Vodnar, Romina Alina Marc, Muhammad Kamran, Muhammad Hamzah Saleem, Abdullah Ahmed Al-Ghamdi, Fahad M. Al-Hemaid, Mohamed S. Elshikh

**Affiliations:** ^1^Department of Horticultural Sciences, Faculty of Agriculture and Environment, The Islamia University of Bahawalpur, Bahawalpur, Pakistan; ^2^Department of Plant Sciences, Quaid-i-Azam University, Islamabad, Pakistan; ^3^Department of Botany, Faculty of Science, Government Sadiq College Women University of Bahawalpur, Bahawalpur, Pakistan; ^4^Department of Agronomy, Faculty of Agriculture and Environment, The Islamia University of Bahawalpur, Bahawalpur, Pakistan; ^5^Faculty of Food Science and Technology, Institute of Life Sciences, University of Agricultural Sciences and Veterinary Medicine of Cluj-Napoca, Cluj-Napoca, Romania; ^6^Food Engineering Department, Faculty of Food Science and Technology, University of Agricultural Sciences and Veterinary Medicine of Cluj-Napoca, Cluj-Napoca, Romania; ^7^School of Agriculture, Food and Wine, The University of Adelaide, Adelaide, SA, Australia; ^8^College of Plant Science and Technology, Huazhong Agricultural University, Wuhan, China; ^9^Department of Botany and Microbiology, College of Science, King Saud University, Riyadh, Saudi Arabia

**Keywords:** plant growth regulators, dormancy, *Solanum tuberosum* L., sprouting, zeatin, g-radiation, cold-treatment, electric current

## Abstract

Development of an efficient and eco-friendly technique to break tuber dormancy in potato (*Solanum tuberosum* L.) is highly demanded due to the production of two or more crops annually. Several physiological and hormonal changes have been found to be related to the breaking of tuber dormancy; however, their consistency with genotypes and different protocols have not been well clarified. This study aims to evaluate the effectiveness of four dormancy-breaking methods, that is, plant growth regulator (PGR) dipping in 30, 60, or 90 mgL^−1^ benzyl amino purine (BAP) and 10, 20, or 30 mgL^−1^ gibberellic acids (GA3) alone and in the combination of optimized concentrations; electric current application at 20, 40, 60, or 80 Vs; cold pre-treatment at 2, 4, or 6 °C; irradiation at 1, 1.5, 2, 2.5, 3, or 3.5 kGy. In addition, changes in endogenous levels of abscisic acid (ABA), zeatin (ZT), and gibberellin A_1_ (GA_1_) in six potato genotypes after subjecting to these methods were investigated. Overall, the highest effective method for dormancy duration was the PGR application which shortened the duration by 18 days, followed by electric current (13 days), cold pre-treatment (9 days), and then irradiation (7 days). The solution of 60 mgL^−1^ BAP significantly reduced the dormancy duration in all genotypes but did not have a significant effect on the sprout length. While 20 mgL^−1^ GA_3_ produced maximum sprout length with a non-significant effect on dormancy duration. The genotype × PGR interaction for dormancy duration was more pronounced in short- and medium-term dormancy genotypes than in long-term dormancy genotypes. The genotypes displayed a significant positive correlation between dormancy duration and ABA levels but exhibited a negative correlation between dormancy duration and ZT as well as GA_1_ levels. From the first to the third week of storage, ABA was decreased in tubers while, however, ZT and GA_1_ were increased. The obtained results could be useful for the postharvest storage of potato tuber and the related field of physiological investigation in future.

## Introduction

Potato is the world's third most popular vegetable in terms of consumption and fourth in terms of production. It is grown on 0.19 million hectares in Pakistan, with an estimated annual production of 4.6 million tons, at an average of 24.2 tons per hectare. Pakistan ranks 19th in terms of production and 12th for export quantity (Bashir et al., 2021). Potato is grown round the year in Pakistan. Autumn (mid-September to January) is the main crop, accounting for 80–85% of total yearly production, with spring (January–May) and summer (April to September) harvests accounting for 10–15% and 1–2%, respectively (Khalid et al., 2020). Its tubers go through a period of dormancy after harvest and are unable to sprout. Thus, the tuber dormancy prevents seed potatoes harvested under the spring and summer crops from being used for subsequent summer and autumn crops (Khan, 2004). Similarly, the autumn-produced seed could also not be used for subsequent spring and summer crops (Haider et al., 2019). The economic dormancy period of seed potatoes varies by country and cropping pattern. With multiple cropping systems in Pakistan, the interval between harvesting and planting consecutive crops is quite short (less than a month). Due to the unavailability of short-term dormancy genotypes, the growers have adapted the autumn-to-autumn cycle by storing the produce of the autumn crop from medium- and long-term dormancy genotypes for the following autumn crop, resulting in seed quality decline (Khan, 2004; Haider et al., 2019).

The dormancy is governed by endogenous levels of abscisic acid (ABA), zeatin (ZT), and gibberellic acid (GA_3_), which are produced through the mevalonic acid pathway (Vivanco and Flores, 2000; Davies, 2004). Their interaction, therefore, has a significant impact on tuber dormancy and sprouting regulation. ABA contents during dormancy are the result of a synthesis-metabolism balance that favors catabolism when dormancy ends (Destefano-Beltrán et al., 2006). The level of ABA is highest in freshly harvested tubers and decreases during postharvest storage. However, there is no specific ABA level below which sprouting begins (Claassens and Vreugdenhil, 2000). Cytokinin levels are low during dormancy and subsequently rise at the initiation of sprouting. The increase in cytokinin content is accompanied by a decrease in ABA during dormancy break (Bryan, 1989). Endogenous GA_1_ was also shown to be rather high just after harvest, decreased during storage, and then increased to its greatest level during vigorous sprout growth (Suttle et al., 2012).

Different approaches have been tested to overcome tuber dormancy in potatoes depending on germplasm and the resources available (Bryan, 1989). Common methods for breaking tuber dormancy include cold shock plus heat (Deligios et al., 2019) and the application of various chemicals such as Rindite (Kim et al., 1999), bromoethane (Alexopoulos et al., 2009), carbon disulfide (Salimi et al., 2010), and thiourea (Mani et al., 2013). They are, however, either ineffective or hazardous to humans and the environment. The use of growth promoters in low quantities is reported to stimulate potato tuber sprouting (Carrera et al., 2000; Alexopoulos et al., 2008). Some growth promoters, such as cytokinin and GA, are also used for bud break and sprouting of seed potatoes and are safe for human use (Wurr, 1978). Previous studies suggest that dormancy break coincides with an abrupt increase in the concentration of ZT and GA_1_ and a decrease in ABA in the buds and adjacent tissues (Suttle, 2004; Hartmann et al., 2011).

The phenomenon of tuber dormancy breakage in potatoes via numerous approaches and consequent physiological changes (changes in oxidative patterns, sugar levels, and protein contents) has been well studied; however, little is known about changes occurring in endogenous levels of hormones in response to individual and combined application of cytokinin, *that is*, BAP, and gibberellin, *that is*, GA_3_, electric current, cold pre-treatment, and irradiation to the tubers of six potato genotypes. The outcomes of this study will provide researchers with new insights into the hormonal regulation of potato tuber dormancy. This study aimed to test the impact of various dormancy-breaking techniques *viz*. plant growth regulators, electric current, cold pre-treatment, and irradiation on tuber dormancy duration of six potato genotypes and observe the endogenous hormonal changes in the tuber.

## Materials and methods

### Plant materials

Six genotypes of potato (*Solanum tuberosum* L.) out of 22 including an equal number of white (FD51-5, Sante, and FD69-1) and red skin genotypes (PRI Red, FD73-49, and FD8-1) were imported from Potato Research Institute (PRI), Sahiwal, Pakistan (Haider et al., 2019). Healthy tubers were selected based on their dormancy behavior excluding the damaged, diseased, or deformed types.

### Application of dormancy-breaking methods

During March 2018, tubers of six potato genotypes were collected after 10 days of harvesting from Potato Research Institute (PRI), Sahiwal. Tubers were dipped in 30, 60, or 90 mgL^−1^ solution of benzylaminopurine (BAP) and 10, 20, or 30 mgL^−1^ solution of gibberellic acid (GA_3_) for 24 h to optimize their concentrations, and for the evaluation of their combined effect on breaking dormancy, the tubers were dipped in each solution for 12 h. For control, tubers were dipped in distilled water. Each treatment was replicated four times, and 30 tubers were used in each replicate, since potato skin is almost impermeable to chemicals (Haider et al., 2019); therefore, parenchyma tissues were exposed to different solutions of PGRs by giving a small cut (15 mm length ×10 mm depth). The tubers, after treatment, were stored under ambient conditions (23.8 ± 1.0°C) till sprouting.

Moreover, tubers were packed in corrugated cardboard boxes and taken to the Post-harvest Research Center, Ayyub Agricultural Research Institute, for cold pre-treatment. The tubers were stored for four days at different storage regimes, *that is*, control, 2°C, 4°C, and 6°C. After cold storage, they were kept at ambient temperature to study sprouting and biochemical characteristics. The temperature and humidity were recorded during ambient storage.

Electric current (20, 40, 60, or 80 V) was applied to tubers for 24 h by penetrating needles (of handmade electric stimulator) 15 mm inside the flesh at the apical and stem ends of the tuber. For control, tubers were injected with needles and an electric current was applied without any voltage. Once treated, the tubers were stored under ambient conditions to calculate different parameters. For the assessment of the influence of gamma rays on tuber hormonal contents, a ^137^Cs source with radiation power of 1 kGy/ 1.5 h was used, at the Nuclear Institute of Agriculture and Biology, Faisalabad. The tubers were exposed to doses of 1, 1.5, 2, 2.5, 3, and 3.5 kGy and stored afterward at ambient temperature for hormonal analysis.

All the above trials were laid out according to completely randomized design (CRD) under factorial settings.

### Data collection

#### Determination of dormancy duration and sprout length

Data on dormancy duration and sprout length were collected from six tubers in each replication on a daily basis. The dormancy was considered to have been broken when sprouts reached 2 mm in length (Van Ittersum and Scholte, 1993; Pande et al., 2007). The length of the sprout was measured using a measuring tape.

#### Determination of endogenous plant growth regulators in tubers

Endogenous plant growth regulators (PGRs) in tubers were analyzed by using the method described by Tang et al. (2011) at one- and three-week storage after treatment of tubers using a Varian ProSTAR 240 high-performance liquid chromatography (HPLC), Walnut Creek, CA, USA, equipped with a Milli-Q ultrapure water purification system. Standards of abscisic acid (ABA) and zeatin (ZT) were obtained from Sigma Chemical, [^2^H_2_]-GA_1_ d_2_-GA_1_ (purity > 90%) was purchased from OlChemim Ltd (Olomouc, Czech Republic), chromatographic purity methanol was from Fisher Chemical, ethanoic acid was analytical grade, and the water used in the experiment was ultrapure.

A thin disk of parenchyma (1 g of tissue) was excised from each tuber, frozen and ground in liquid nitrogen, homogenized, and then extracted overnight with 30 ml of 80% cold aqueous methanol (<0 °C) in darkness at 4°C. The extracts were purified by a protocol adapted by Bensen et al. (1990). After filtration, the extracts were reduced to the aqueous phase. Each extract obtained was then centrifuged at 5,000 rpm and 4°C for 15 min, and the supernatant was collected. Then, fresh cold methanol was poured onto the remnant and extracted three times as stated above. The total methanolic extract was dried in a rotary evaporator (Laborata 4002, Heidolph, Nürnberg, Germany) and dissolved in 10 ml methanol. The extracts were fractionated using a C-18 chromatographic column (150 ×4.6 mm, 5 μm) and a mixture of acetonitrile (Solvent A)/1 percent (v/v) acetic acid containing 0.5 ml liter^−1^ triethylamine (Solvent B). The following were the solvent gradient conditions: initial: 5% A, hold for 10 min, a linear gradient to 30% A in 15 min, hold for 35 min, a linear gradient to 100% A, hold for 3 min, return to initial conditions in 5 min. The column temperature was 35°C, flow rate of 1 ml min^−1^, and UV detection wavelength of 254 nm. ABA, ZT, and GA_1_ were measured by injection of the extract into a reverse phase HPLC. Determinations of ABA, ZT, and GA_1_ were performed on the same sample and expressed in ng g^−1^ FW.

### Statistical analyses

All data were subjected to a three-way analysis of variance (treatment × genotype × storage period) under three factors factorial completely randomized design (CRD) using Statistix9^®^ software (Analytical Software, Tallahassee, USA), and the results are interpreted as the relative contribution of treatment, genotype, storage period, and their interactions by determining the percentage of total variance from the corresponding sum of squares (SS) as performed by Kyriacou et al. (2021). The least significant difference (LSD) test was used to compute mean comparisons and main effects for treatment, genotype, storage period, and their interactions at P ≤ 0.05. The SPSS 16.0 program was used to conduct Pearson's correlation analysis (USA). The correlation was analyzed by the general linear model procedure in SAS, version 9.2 (Cary, NC).

## Results

### Screening trial

There were significant differences in the duration of postharvest dormancy among the original 22 genotypes evaluated in the screening trial (Haider et al., 2019). From these, three distinct groups were chosen based on their tuber sprouting behavior. Short-term dormancy genotypes included FD51-5 and PRI Red; genotypes with medium-term dormancy included Sante and FD73-49, while FD69-1 and FD8-1 were classified as long-term dormancy genotypes. Each group contained white and red skin genotypes as both colors of tubers are equally liked and consumed by the consumers in the country (Pakistan).

### Individual application of PGRs and their optimization

#### Effect on tuber dormancy and sprout length

**Figure 1:**
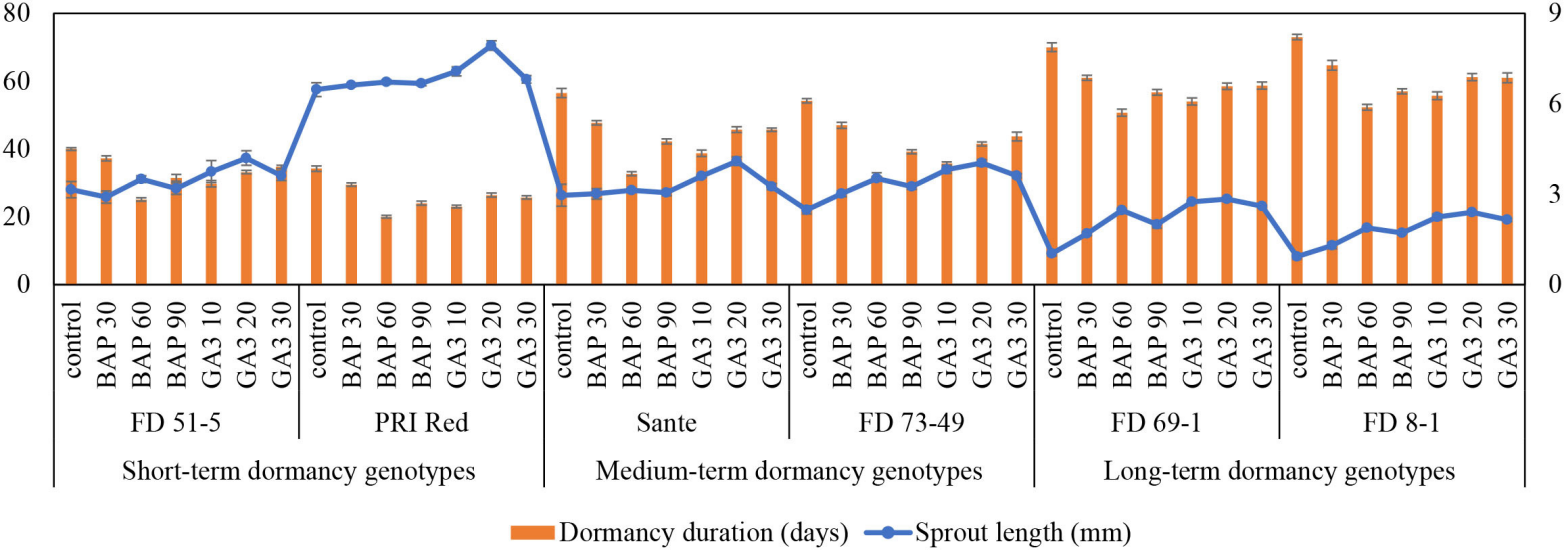
Mean comparison of dormancy duration (days) and sprout length (mm) in potato tubers under genotype × PGR interactive effect. Vertical error bars represent standard error of means.

PGRs, genotypes, and their interactive effect (PGRs × genotypes) were significant (*P* ≤ 0.05) for dormancy duration and sprout length (Table 1A). Among PGRs, 60 mgL^−1^ was the most effective dose of BAP in shortening the dormancy duration and 20 mgL^−1^ of GA_3_ in increasing the sprout length, in all genotypes. The interactive effect was more pronounced in the short-term (PRI Red and FD51-5) and medium-term (FD73-49 and Sante) dormancy genotypes and less pronounced in the long-term (FD69-1 and FD8-1) dormancy genotypes (Figure 1).

**Table 1A:** Mean comparison of dormancy duration and sprout length of potato tubers influenced by PGRs and genotypes during the year 2018.

**Factors**		**Dormancy duration (days)**	**Sprout length (mm)**
Treatment (T)	Control	53.5a	2.73f
	30 mgL^−1^ BAP	46.7b	3.09e
	60 mgL^−1^ BAP	34.3f	3.47c
	90 mgL^−1^ BAP	40.6d	3.21d
	10 mgL^−1^ GA_3_	38.2e	3.77b
	20 mgL^−1^ GA_3_	43.1c	4.15a
	30 mgL^−1^ GA_3_	43.9c	3.57c
LSD T (*P* ≤ 0.05)		**0.95**	**0.141**
Genotype (G)	FD51-5	32.1e	3.35b
	PRI Red	25.0f	6.80a
	Sante	43.2c	3.20c
	FD73-49	41.0d	3.29bc
	FD69-1	57.5b	2.10d
	FD8-1	59.7a	1.70e
LSD G (*P ≤* 0.05)		**0.88**	**0.130**
LSD T × G (*P ≤* 0.05)		2.386	0.370

*BAP, benzylaminopurine; GA_3_, gibberellic acid.*

*Treatment means followed by the same letter are not significantly different.*

*LSD, least significant difference*.

#### Effect on tuber endogenous plant growth regulators

Abscisic acid (ABA), zeatin (ZT), and gibberellin A_1_ (GA_1_) in experimental tubers were significantly influenced by the dormancy-breaking methods, genotypes, and storage periods and the two-way interaction of genotype × storage period. The rest of the interactions were non-significant (Table 1B). All the treated tubers resulted in significantly lower ABA and higher ZT, and GA_1_ compared to the control. There was a significant positive correlation (r ≥ 0.99) between dormancy and ABA levels. The short dormancy genotypes (FD51-5 and PRI Red) had significantly lower levels of ABA than the moderate (Sante and FD73-49) and long-term dormancy genotypes (Sante and FD73-49), and the moderate dormancy genotypes had lower levels of ABA than the long-dormancy genotypes. There was a significant negative correlation between dormancy and ZT levels (r ≥ −0.94) and dormancy and GA_1_ levels (r ≥ −0.94). The short-term dormancy genotypes had significantly higher levels of ZT and GA_1_ than those of the moderate- and long-dormancy genotypes, and the medium-term dormancy genotypes had significantly higher levels of ZT and GA_1_ than the long-term dormancy genotypes.

**Table 1B:** Periodic changes in the contents of ABA, ZT, and GA_1_ in experimental tubers of six potato genotypes dipped in PGRs during 2018.

**Factors**		**ABA**	**ZT**	**GA_1_**
		**ng g^−1^ FW**	**ng g^−1^ FW**	**ng g^−1^ FW**
Treatment (T)	Control	98.2a	16.1c	0.52c
	30 mgL^−1^ BAP	97.4a	16.2c	0.53bc
	60 mgL^−1^ BAP	90.0d	16.9a	0.58a
	90 mgL^−1^ BAP	93.9c	16.4b	0.55ab
	10 mgL^−1^ GA_3_	95.1bc	16.3bc	0.56ab
	20 mgL^−1^ GA_3_	95.2b	16.3bc	0.58a
	30 mgL^−1^ GA_3_	95.5b	16.2bc	0.55b
LSD T (*P ≤* 0.05)		**1.27**	**0.22**	**0.023**
Genotype (G)	FD51-5	62.0e	19.1b	0.70b
	PRI red	54.1f	22.9a	0.84a
	Sante	100.7c	14.3d	0.47c
	FD73-49	95.1d	17.0c	0.45cd
	FD69-1	122.6b	12.7e	0.44d
	FD8-1	135.9a	12.2f	0.39e
LSD G (*P ≤* 0.05)		**1.18**	**0.20**	**0.021**
Storage period (SP)	1 Week	125.7a	9.7a	0.59a
	3 Week	64.4b	23.0b	0.51b
LSD SP (*P ≤* 0.05)		**0.68**	**0.12**	**0.012**
LSD T × G (*P ≤* 0.05)		NS	NS	NS
LSD T × SP (*P ≤* 0.05)		NS	NS	NS
LSD G × SP (*P ≤* 0.05)		1.67	0.29	0.030
LSD T × G × SP (*P ≤* 0.05)		NS	NS	NS

*NS, non-significant at P ≤ 0.05.*

*Treatment means sharing same letter differ non-significantly.*

*ABA, abscisic acid; GA_1_, gibberellin A_1_; BAP, benzylaminopurine; GA_3_, gibberellic acid; LSD, least significant difference*.

**Figure 2:**
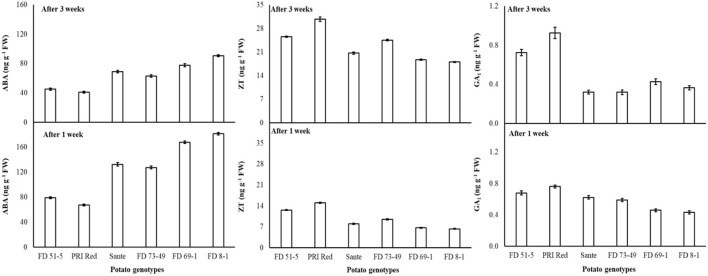
Change in ABA, ZT, and GA_1_ levels in tuber during storage period (3 weeks) following treatment with alone benzylaminopurine and gibberellic acid. Vertical error bars represent standard error of means (n: 4).

Under the genotype × storage period interaction, ABA decreased more rapidly in the long-dormancy genotypes than in the moderate or short dormancy genotypes (Figure 2). The rates of increase in ZT between week one and week three were similar in four of the genotypes (FD51-5, PRI Red, Sante, and FD73-49) (Figure 2). GA_1_ increased between week one and week three in the short dormancy genotypes but decreased in the moderate- to long-dormancy genotypes (Figure 2).

### Individual and combined application of optimized doses of PGRs

#### Effect on tuber dormancy and sprout length

**Figure 3:**
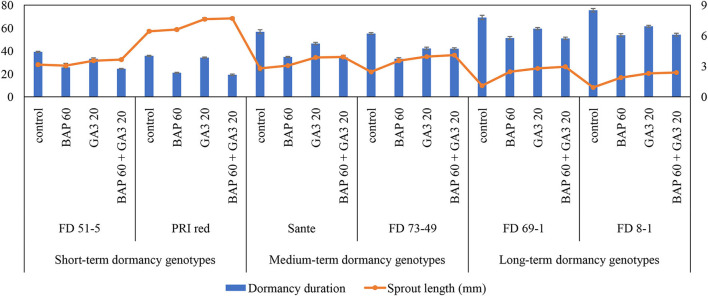
Mean comparison of dormancy duration (days) and sprout length (mm) in potato tubers under genotype × PGR interactive effect. Vertical error bars represent standard error of means.

The individual effects of PGRs and genotypes and their interaction were significant (*P* ≤ 0.05) for shortening the dormancy duration (Table 2A). Among PGRs, 60 mgL^−1^ BAP and its combination with 20 mgL^−1^ GA_3_ effectively reduced the dormancy duration by 18.6 and 17.7 days and increased the sprout length by 1.2 and 1.5 times, respectively (Table 2A). “PRI Red” displayed the shortest (27.5 days) and “FD8-1” displayed the longest dormancy duration (Table 2A). The interaction was more pronounced in the short- (PRI Red and FD51-5) and medium-term (FD73-49 and Sante) dormancy genotypes and less pronounced in the long-term (FD8-1 and FD69-1) dormancy genotypes (Figure 3).

**Table 2A:** Mean comparison of dormancy duration and sprout length of potato tubers dipped in optimized doses of PGRs and genotypes during 2019.

**Factors**		**Dormancy duration (days)**	**Sprout length (mm)**
Treatment (T)	Control	55.2a	2.81c
	60 mgL^−1^ BAP	36.6c	3.43b
	20 mgL^−1^ GA_3_	46.2b	4.01a
	60 mgL^−1^ BAP + 20 mgL^−1^ GA_3_	37.5c	4.12a
LSD T (*P ≤* 0.05)		**1.18**	**0.142**
Genotype (G)	FD51-5	30.6d	3.35b
	PRI Red	27.5e	7.09a
	Sante	43.1c	3.41b
	FD73-49	43.1c	3.51b
	FD69-1	57.7b	2.33c
	FD8-1	61.2a	1.87d
LSD G (*P ≤* 0.05)		**1.45**	**0.174**
LSD T × G (*P ≤* 0.05)		2.901	0.348

*BAP, benzylaminopurine; GA_3_, gibberellic acid.*

*Treatment means followed by the same letter are not significantly different.*

*BAP, benzylaminopurine; GA_3_, gibberellic acid; LSD, least significant difference*.

#### Effect on tuber endogenous plant growth regulators

**Figure 4:**
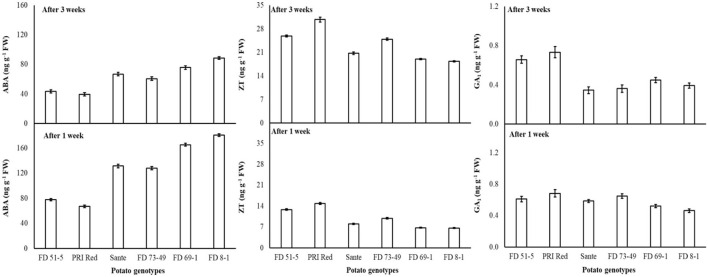
Change in ABA, ZT, and GA_1_ levels in tuber during storage period (3 weeks) following treatment with benzylaminopurine and gibberellic acid together. Vertical error bars represent standard error of means (n: 4).

The lowest ABA (89.9 ng g^−1^ FW) and highest ZT (17.0 ng g^−1^ FW) and GA_1_ (0.53 ng g^−1^ FW) levels were found with a combined application of 60 mgL^−1^ of BAP and 20 mgL^−1^ GA_3_. The maximum ABA was observed in FD8-1, while the minimum was observed in PRI Red (Table 2B). Levels of ABA (125.7ng g^−1^ FW) and GA_1_ (0.58 ng g^−1^ FW) at week one were significantly higher than ABA (64.4ng g^−1^ FW) and GA_1_ (0.49 ng g^−1^ FW) at week three (Table 2B). ZT was significantly lower in week one (9.7ng g^−1^ FW) than in week three (23.0ng g^−1^ FW) (Table 2B). A significant positive correlation (r = 0.99) was observed in genotypes between dormancy and ABA levels. The short-term dormancy genotypes (PRI Red and FD51-5) had significantly lower levels of ABA than the medium-term (FD73-49 and Sante) and long-term dormancy genotypes (FD8-1 and FD69-1), and the medium-term dormancy genotypes had relatively lower levels of ABA than the long-term dormancy genotypes. There was a significant negative correlation between dormancy and ZT levels (r ≥ −0.94) and dormancy and GA_1_ levels (r ≥ −0.94). The rates of increase in ZT between week 1 and week 3 were greater in genotypes PRI Red and FD73-49 (Figure 4). However, GA_1_ increased during the storage time in the short-term dormancy genotypes but decreased in the medium- to long-term dormancy genotypes (Figure 4).

**Table 2B:** Periodic changes in the contents of ABA, ZT, and GA_1_ in experimental tubers of six potato genotypes dipped in optimized doses of PGRs during 2019.

**Factors**		**ABA**	**ZT**	**GA_1_**
		**ng g^−1^ FW**	**ng g^−1^ FW**	**ng g^−1^ FW**
Treatment (T)	Control	98.5a	16.1c	0.49c
	60 mgL^−1^ BAP	91.1c	16.8b	0.57a
	20 mgL^−1^ GA_3_	95.2b	16.3c	0.53b
	60 mgL^−1^ BAP + 20 mgL^−1^ GA_3_	89.9c	17.0a	0.57a
LSD T (*P ≤* 0.05)		**1.25**	**0.22**	**0.026**
Genotype (G)	FD51-5	60.4e	19.4b	0.63b
	PRI red	53.3f	22.8a	0.71a
	Sante	99.0c	14.4d	0.46d
	FD73-49	94.3d	17.4c	0.51c
	FD69-1	120.5b	12.8e	0.48cd
	FD8-1	134.6a	12.5f	0.43e
LSD G (*P ≤* 0.05)		**1.53**	**0.27**	**0.032**
Storage period (SP)	1 Week	124.9a	9.8a	0.58a
	3 Week	62.3b	23.3b	0.49b
LSD SP (*P ≤* 0.05)		**0.89**	**0.16**	**0.019**
LSD T × G (*P ≤* 0.05)		NS	NS	NS
LSD T × SP (*P ≤* 0.05)		NS	NS	NS
LSD G × SP (*P ≤* 0.05)		2.17	0.39	0.046
LSD T × G × SP (*P ≤* 0.05)		NS	NS	NS

*NS, non-significant at P ≤ 0.05.*

*Treatment means sharing same letter differ non-significantly.*

*ABA, abscisic acid; GA_1_, gibberellin A_1_; BAP, benzylaminopurine; GA_3_, gibberellic acid; LSD, least significant difference*.

### Electric shock of tubers

#### Effects on tuber dormancy and sprout length

The effect of electric current and genotype was significant (*P* ≤ 0.05) for dormancy duration and sprout length in tubers during both years (Table 3A), but their interactive effect was found significant only for the year 2019 (Table 3B). In both years, the shortest dormancy duration was observed in tubers treated with the electric current at 80 V which was significantly different from the control. PRI Red showed the shortest dormancy duration.

**Table 3A:** Mean comparison of dormancy duration and sprout length of six genotypes potato tubers during 2018–2019 underwent direct electric current.

**Factors**		**Dormancy duration (days)**		**Sprout length (mm)**	
		**2018**	**2019**	**2018**	**2019**
Electric current (Ec)	0-V	54.0a	53.7a	2.75b	2.70c
	20-V	49.7b	49.6b	2.79b	2.78bc
	40-V	48.1c	48.1c	2.79b	2.85bc
	60-V	46.4d	46.1d	2.84b	2.90b
	80-V	41.0e	40.6e	3.52a	3.51a
LSD T (*P ≤* 0.05)		**1.36**	**1.35**	**0.179**	**0.156**
Genotype (G)	FD51-5	36.9d	36.5d	3.19b	3.19b
	PRI Red	30.7e	31.0e	6.46a	6.53a
	Sante	48.0c	48.6c	2.98c	2.93c
	FD73-49	48.2c	47.8c	2.64d	2.66d
	FD69-1	60.2b	59.3b	1.26e	1.30e
	FD8-1	63.2a	62.7a	1.09e	1.08f
LSD G (*P ≤* 0.05)		**1.50**	**1.47**	**0.196**	**0.171**
LSD Ec × G (*P ≤* 0.05)	NS	3.308	NS	NS

*NS, non-significant at P ≤ 0.05.*

*Treatment means followed by the same letter are not significantly different.*

*LSD, least significant difference*.

**Table 3B:** Mean comparison of dormancy duration of potato in 2019 under direct electric current and genotype interactive effects.

**Treatments (T)**	**Genotypes (G)**					
	**FD51-5**	**PRI Red**	**Sante**	**FD73-49**	**FD69-1**	**FD8-1**
0-V	41.0k	33.7mn	55.2ef	53.7fg	68.7a	69.7a
20-V	38.2kl	32.2no	50.5ghi	51.2gh	62.0bc	63.5b
40-V	38.2kl	31.5no	49.0hij	48.5hij	58.5de	63.2b
60-V	35.7lm	31.5no	47.7ij	46.0j	56.7def	59.2cd
80-V	29.2op	26.2p	40.5k	39.5k	50.7ghi	57.7de

*LSD Ec × G (P ≤ 0.05) = 3.308.*

*Treatment means followed by the same letter are not significantly different.*

*LSD, least significant difference*.

#### Effect on tuber endogenous plant growth regulators

**Figure 5:**
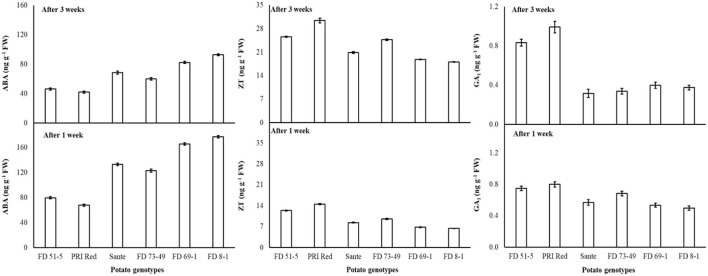
Change in ABA, ZT, and GA_1_ levels in tuber during storage period (3 weeks) following treatment with low temperature under cold storage. Vertical error bars represent standard error of means (n: 4).

The contents of ABA, ZT, and GA_1_ for electric current, genotypes, and storage periods are given in Table 3C. ABA decreased with increasing voltages. There were no significant differences for ZT at most voltages but were significantly higher at the 60 V and 80 V. GA_1_ also increased with increasing voltage. ABA (126.1 ng g^−1^ FW) and GA_1_ (0.62 ng g^−1^ FW) were significantly higher in week one than ABA (64.7 ng g^−1^ FW) and GA_1_ (0.51 ng g^−1^ FW) in week three. ZT was significantly lower in week one (9.7 ng g^−1^ FW) than in week three (23.0 ng g^−1^ FW). There was a significant positive correlation (r = 0.99) between dormancy and ABA levels in genotypes. The short-term dormancy genotypes had significantly lower levels of ABA than the moderate- and long-term dormancy genotypes, whereas a negative correlation was observed between dormancy and ZT (r = −0.96) and GA_1_ (r = −0.92). The genotypes PRI Red and FD51-5 had higher levels of GA_1_ than FD73-49 and Sante which had higher levels of GA_1_ than FD8-1 and FD69-1 (Figure 5).

**Table 3C:** Periodic changes in the contents of ABA, ZT, and GA_1_ in experimental tubers of six potato genotypes following treatment with direct electric current during the year 2018–2019.

**Factors**		**ABA**	**ZT**	**GA_1_**
		**ng g^−1^ FW**	**ng g^−1^ FW**	**ng g^−1^ FW**
Electric current (Ec)	0-V	98.9a	16.2c	0.51c
	20-V	97.8a	16.2c	0.53c
	40-V	95.8b	16.2c	0.56b
	60-V	94.0c	16.5b	0.58b
	80-V	90.4d	16.8a	0.64a
LSD T (*P ≤* 0.05)		**1.21**	**0.21**	**0.026**
Genotype (G)	FD51-5	62.9e	18.9b	0.72b
	PRI red	55.1f	22.6a	0.82a
	Sante	101.2c	14.6d	0.46d
	FD73-49	94.6d	17.2c	0.50c
	FD69-1	122.5b	12.7e	0.45d
	FD8-1	136.0a	12.3f	0.43d
LSD G (*P ≤* 0.05)		**1.32**	**0.23**	**0.029**
Storage period (SP)	1 Week	126.1a	9.7b	0.62a
	3 Week	64.7b	23.0a	0.51b
LSD SP (*P ≤* 0.05)		**0.76**	**0.13**	**0.017**
LSD Ec × G (*P ≤* 0.05)		NS	NS	NS
LSD Ec × SP (*P ≤* 0.05)		NS	NS	NS
LSD G × SP (*P ≤* 0.05)		1.87	0.32	0.041
LSD Ec × G × SP (*P ≤* 0.05)		NS	NS	NS

*NS, non-significant at P ≤ 0.05.*

*Treatment means sharing same letter differ non-significantly.*

*LSD, least significant difference*.

### Cold pre-treatment of tubers

#### Effect on tuber dormancy and sprout length

The dormancy duration and sprout length were significantly (*P* < 0.05) influenced by the storage temperatures and the genotypes in both years (Table 4A). The lowest dormancy duration was recorded in tubers stored at 2°C in both years, and the longest dormancy duration was found in tubers of control. The shortest dormancy duration and highest sprout length were recorded in “PRI Red” in both years. The interactive effect of storage temperature and genotype did not have a significant effect on the dormancy duration but on the sprout length (Table 4A).

**Table 4A:** Mean comparison of dormancy duration and sprout length of six potato genotypes tubers during 2018–2019 underwent low-temperature cold storage.

**Factors**		**Dormancy duration (days)**		**Sprout length (mm)**	
		**2018**	**2019**	**2018**	**2019**
Temperature (T)	Control	54.7a	53.7a	2.72c	2.76c
	2°C	45.5d	44.7d	3.38a	3.36a
	4°C	48.5c	48.3c	2.95b	2.98b
	6°C	51.0b	50.9b	2.81bc	2.82bc
LSD T (*P ≤* 0.05)		**1.16**	**1.34**	**0.196**	**0.164**
Genotype (G)	FD51-5	38.1c	37.2d	3.14b	3.164b
	PRI Red	32.8d	32.4e	6.39a	6.34a
	Sante	50.6b	48.6c	2.99b	3.00b
	FD73-49	49.2b	47.8c	2.66c	2.70c
	FD69-1	63.9a	64.0b	1.34d	1.41d
	FD8-1	64.8a	66.6a	1.26d	1.26d
LSD G (*P ≤* 0.05)		**1.42**	**1.64**	**0.240**	**0.201**
LSD T × G (*P ≤* 0.05)	NS	NS	NS	NS

*NS, non-significant at P ≤ 0.05.*

*Treatment means followed by the same letter are not significantly different.*

*LSD, least significant difference*.

#### Effect on tuber endogenous plant growth regulators

**Figure 6:**
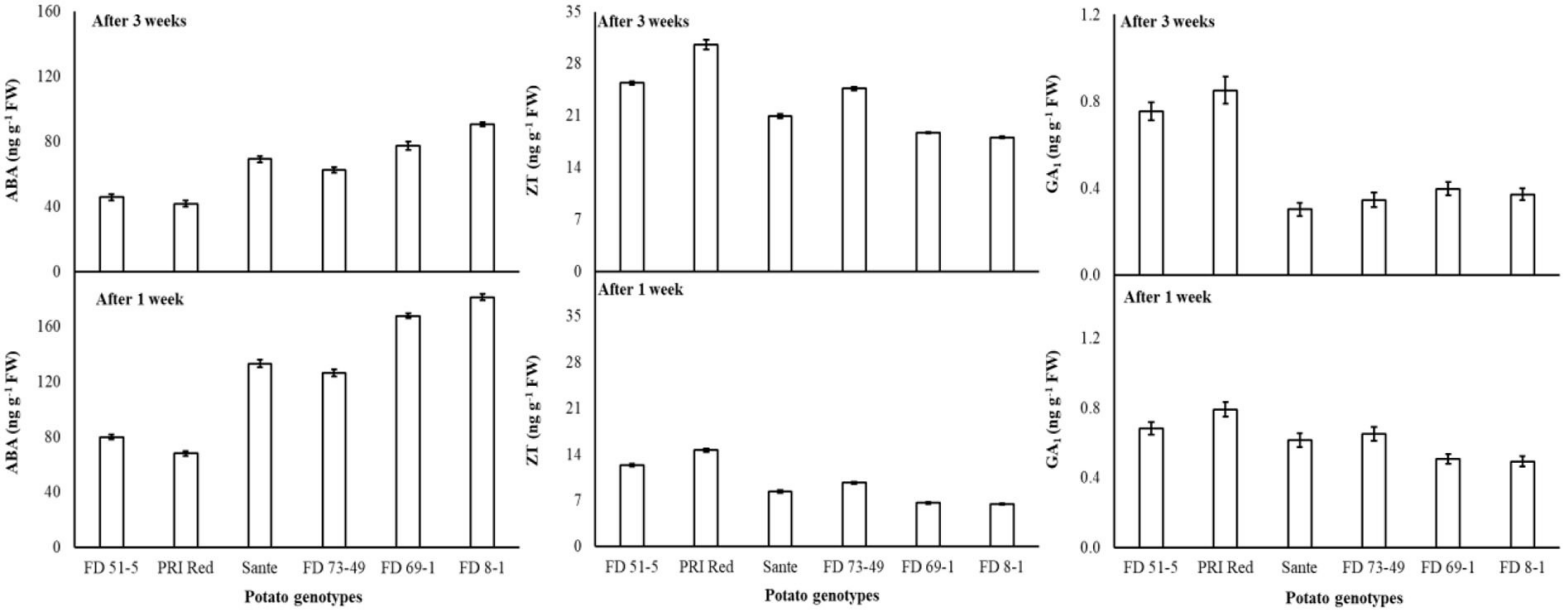
Change in ABA, ZT, and GA_1_ levels in tuber during storage period (3 weeks) following treatment with electric current. Vertical error bars represent standard error of means (n: 4).

ABA levels decreased with the lowest temperature (2°C) treatment. There were no significant differences for ZT at most storage temperatures, with the exception that ZT was significantly higher at the lowest temperature. GA_1_ also increased with the lowermost temperature. FD8-1 had the highest ABA content (135.0 ng g^−1^ FW) and the lowest ZT (12.0 ng g^−1^ FW) and GA_1_ (0.43 ng g^−1^ FW) of the genotypes studied, while PRI Red had the lowest ABA (54.9 ng g^−1^ FW) and the highest ZT (22.5 ng g^−1^ FW) and GA_1_ (0.89 ng g^−1^ FW) contents (Table 4B). ABA (124.4 ng g^−1^ FW) and GA_1_ (0.64 ng g^−1^ FW) were significantly higher in week one than ABA (65.2 ng g^−1^ FW) and GA_1_ (0.54 ng g^−1^ FW) contents in week three (Figure 6). On the contrary, ZT was significantly lower in week one (9.7 ng g^−1^ FW) than in week three (23.0 ng g^−1^ FW) (Figure 6).

**Table 4B:** Periodic changes in the contents of ABA, ZT, and GA_1_ in experimental tubers of six potato genotypes following treatment with low-temperature cold storage during 2018–2019.

**Factors**		**ABA**	**ZT**	**GA_1_**
		**ng g^−1^ FW**	**ng g^−1^ FW**	**ng g^−1^ FW**
Temperature (T)	Control	97.9a	16.1b	0.54d
	2°C	91.2d	16.6a	0.65a
	4°C	93.8c	16.4b	0.60b
	6°C	96.2b	16.2b	0.57c
LSD T (*P ≤* 0.05)		**0.97**	**0.21**	**0.025**
Genotype (G)	FD51-5	62.9e	18.9b	0.79
	PRI red	54.9f	22.5a	0.89
	Sante	100.8c	14.6d	0.44
	FD73-49	91.4d	17.1c	0.51
	FD69-1	123.9b	12.8e	0.47
	FD8-1	135.0a	12.2f	0.43
LSD G (*P ≤* 0.05)		**1.18**	**0.26**	**0.030**
Storage period (SP)	1 Week	124.4a	9.7b	0.64
	3 Week	65.2b	23.0a	0.54
LSD SP (*P ≤* 0.05)		**0.68**	**0.15**	**0.017**
LSD T × G (*P ≤* 0.05)		2.368	NS	NS
LSD T × SP (*P ≤* 0.05)		NS	NS	NS
LSD G × SP (*P ≤* 0.05)		1.67	0.37	0.043
LSD T × G × SP (*P ≤* 0.05)		NS	NS	NS

*NS, non-significant at P ≤ 0.05.*

*Treatment means sharing same letter differ non-significantly.*

*LSD, least significant difference*.

### Irradiation of tubers

#### Effect on tuber dormancy and sprout length

γ-radiation significantly influenced the dormancy duration of tubers of different potato genotypes in both years (Table 5A). However, the interactive effect of irradiation dose × genotype did not significantly alter the dormancy duration. The lowest dormancy duration in 2018 (48.4 days) and 2019 (48.9 days) was recorded in the tubers treated with the highest dose of radiation (3.5 kGy) compared to the highest dormancy duration in 2018 (55.9 days) and 2019 (55.5 days) was found in the tubers of control. Among genotypes, PRI Red showed the shortest dormancy duration during both years 2018 (34.8 days) and 2019 (35.9 days), and FD8-1 showed the longest in both 2018 (68.9 days) and 2019 (68.7 days).

**Table 5A:** Mean comparison of dormancy duration and sprout length of potato tubers for two years (2018–2019) under γ-irradiation doses and genotype main effects.

**Factors**		**2018**		**2019**	
		**Dormancy duration**	**Sprout length**	**Dormancy duration**	**Sprout length**
		**(Days)**	**(mm)**	**(Days)**	**(mm)**
Irradiation dose (ID)	Control	55.9a	2.8d	55.46a	2.80c
	1.0 kGy	55.0ab	2.8cd	55.29a	2.84c
	1.5 kGy	54.3b	2.8cd	54.33ab	2.86c
	2.0 kGy	53.8bc	2.8cd	53.92bc	2.87bc
	2.5 kGy	52.7c	2.9bc	52.88cd	2.92bc
	3.0 kGy	51.5d	3.0b	52.00d	3.01b
	3.5 kGy	48.3e	3.2a	48.88e	3.23a
LSD ID (*P ≤* 0.05)	**1.15**	**0.14**	**1.219**	**0.143**
Genotypes (G)	FD51-5	40.4e	3.2b	38.07d	3.19b
	PRI Red	34.8f	6.7a	35.96e	6.61a
	Sante	54.7c	2.9c	55.07b	2.89c
	FD73-49	52.3d	2.6d	52.72c	2.63d
	FD69-1	67.5b	1.2e	69.00a	1.23e
	FD8-1	68.9a	1.0e	68.68a	1.054f
LSD G (*P ≤* 0.05)	**1.06**	**0.13**	**1.128**	**0.132**
LSD ID × G (*P ≤* 0.05)	NS	NS	NS	NS

*NS, non-significant at P ≤ 0.05.*

*Treatment means followed by the same letter are not significantly different.*

*LSD, least significant difference*.

#### Effect on tuber endogenous plant growth regulators

**Figure 7:**
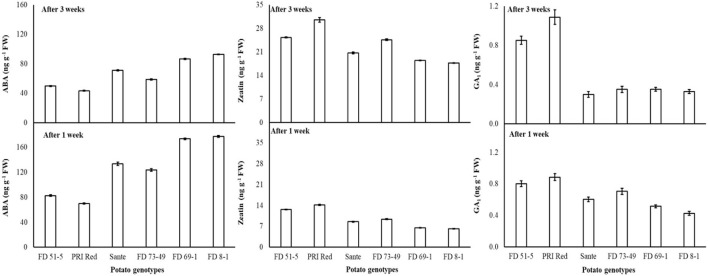
Change in ABA, ZT, and GA_1_ levels in tuber during storage period (3 weeks) following treatment with γ-irradiation. Vertical error bars represent standard error of means (n: 4).

The concentration of ABA decreased with increasing radiation dosage (Table 5B). There were no significant differences in ZT contents at most dosage levels, with the exception that ZT was significantly higher at the highest dosage than at the other six lower doses (Table 5B). GA_1_ increased with increasing dosage. In general, GA_1_ was significantly higher when differences in dosage were greater than 1.0–1.5 KGy (Table 5B). ABA and GA_1_ decreased from week one to week three, while ZT increased (Table 5B). There was a significant positive correlation (r = 0.99) between dormancy and ABA levels and negative correlation (r = −0.94) between dormancy and ZT (-0.97) and GA_1_ (-0.94) levels. ABA decreased more rapidly in the long-dormancy genotypes than in the moderate or short dormancy genotypes, while ZT increased between week one and week three (Figure 7). The rate of increase was prominent in PRI Red and FD73-49 (Figure 7). GA_1_ increased between week one and week three in the short dormancy genotypes but decreased in the moderate- to long-dormancy genotypes (Figure 7).

**Table 5B:** Periodic changes in the contents of ABA, ZT, and GA_1_ in experimental tubers of six potato genotypes following γ-radiation during 2018–2019.

**Factors**		**ABA**	**Zeatin**	**GA_1_**
		**ng g^−1^ FW**	**ng g^−1^ FW**	**ng g^−1^ FW**
Irradiation (I)	Control	98.2a	16.1b	0.55e
	1.0 kGy	97.8a	16.1b	0.57de
	1.5 kGy	97.5ab	16.2b	0.58cd
	2.0 kGy	97.1ab	16.2b	0.58cd
	2.5 kGy	96.6bc	16.2b	0.60bc
	3.0 kGy	96.0cd	16.3ab	0.63b
	3.5 kGy	94.9d	16.5a	0.69a
LSD I (*P ≤* 0.05)		**1.09**	**0.22**	**0.025**
Genotype (G)	FD51-5	66.2e	18.9b	0.83b
	PRI red	56.6f	22.3a	0.98a
	Sante	102.3c	14.6d	0.45d
	FD73-49	91.1d	17.0c	0.53c
	FD69-1	129.9b	12.5e	0.43d
	FD8-1	135.2a	11.9f	0.38e
LSD G (*P ≤* 0.05)		**1.01**	**0.20**	**0.023**
Storage period (SP)	1 Week	126.8a	9.5b	0.66a
	3 Week	67.0b	22.9a	0.55b
LSD SP (*P ≤* 0.05)		**0.58**	**0.12**	**0.013**
LSD I × G (*P ≤* 0.05)		NS	NS	NS
LSD I × SP (*P ≤* 0.05)		NS	NS	NS
LSD G × SP (*P ≤* 0.05)		1.42	0.29	0.032
LSD I × G × SP (*P ≤* 0.05)		NS	NS	NS

*NS, non-significant at P ≤ 0.05.*

*Treatment means sharing same letter differ non-significantly.*

*LSD, least significant difference*.

## Discussion

The seed potato sector in Pakistan requires genotypes with varied tuber dormancy lengths to fit into various cropping calendars of the growers (Haider et al., 2019). Commonly, the use of dormancy-breaking methods could increase the flexibility in raising consecutive crops. The dormancy breakage through the use of chemicals including bromoethane, thiourea, and rindite has been widely studied, but these chemicals are harmful and unsafe for humans and the environment. Therefore, developing an efficient, safe, and eco-friendly method for the breakage of seed-tuber dormancy is highly demanded.

It has been reported that the reduction of the dormancy period might be due to the effect of benzylaminopurine (BAP) on the nutrient sink that is essential in maintaining the G_1_-S and G_2_-M transitions in the plant cell. The exogenous application of cytokinin affects the endogenous nutrient and zeatin (ZT) contents and eventually results in the release of dormancy (Hannapel et al., 2004). During dormancy breaking, the endogenous level of ZT increased, and by contrast, the level of ABA decreased (Deligios et al., 2019). In this study, it was found that the application of 60 mg L^−1^ BAP significantly reduced the dormancy duration of potato tubers by 19.1 days when compared to the untreated control, and it approved the significant role of exogenous cytokinin in dormancy breaking. The response to treatments for breaking tuber dormancy was highly affected by genotypes; hence, an optimized concentration for each genotype has practical usage for growers.

The combined application of benzyl adenine (BA) with gibberellic acid (GA_3_) could significantly enhance sprouting when compared to the same levels of BA or GA applied individually (Njogu et al., 2015). The exogenously applied GA_3_ could stimulate the sprouting of excised tuber buds by enhancing the level of endogenous GA (Alexopoulos et al., 2007; Hartmann et al., 2011). Among different types of GA applied through an exogenous injection, GA_1_ was the only effective to terminate the dormancy of shortly stored tubers (Suttle, 2004). The endogenous levels of PGRs have been investigated, and it was found that the ABA level decreased and, by contrast, ZT and GA_1_ increased when the storage duration was prolonged (Suttle, 1998). In this study, the endogenous levels of ABA, ZT, and GA_1_ in the tubers treated with combined application of BAP and GA_3_ were significantly changed from the first week to the third week when compared to the control. These changes were found to be more evident in long-term dormancy genotypes than in medium- or short-term dormancy genotypes. However, either a decrease in dormancy duration or an increase in sprout length was more pronounced in short- and medium-term dormancy genotypes. Hence, it was suggested that there is an obvious difference among genotypes in hormonal changes during the dormancy-breaking period.

The exogenous application of GA_3_ enhances the endogenous level of GAs which affects the synthesis of amylase which enhances starch break down causing quick sprout growth (Krochko et al., 1998; Zhang et al., 2014). It was suggested that in this study, the increase in sprout length in the tubers treated with 20 mgL^−1^ GA_3_ might be due to the process of starch breakdown mediated by the enzyme, amylase (Sergeeva et al., 2012; Dai et al., 2016; Xie et al., 2018). In the subsequent year, the best doses of BAP and GA_3_ were experimented together to determine their combined effect on dormancy period and sprout length. The dormancy duration was statistically similar to that of individually applied BAP, but the combined application had a prominent effect on sprout length of tubers compared to individual applications of BAP and GA_3_.

It is thought that ABA is metabolized mainly by oxidation of the 8′ methyl group to form the unstable intermediate 8′-hydroxy-ABA, which spontaneously rearranges to form phaseic acid (PA), which is then reduced to form dihydrophaseic acid (DPA) (Krochko et al., 1998; Alexopoulos et al., 2006; Wang et al., 2020), while ZT level increased with progression in storage (Letham and Palni, 1983; Horgan, 1986; Alexopoulos et al., 2006). Our findings are consistent with these, as we observed a gradual decrease in the endogenous level of ABA and an increase in the level of ZT. With the increase in storage period (from first to the third week), ZT and GA_1_ increased in the short dormancy genotypes and ABA decreased; however, GA_1_ decreased in the moderate- to long-dormancy genotypes.

The variations observed in dormancy duration and sprout length of different genotypes under the effect of electric current are in line with the findings of Kocacaliskan et al. (1989), who observed the decrease in dormancy duration and increase in sprout length with the passage of storage period following application of electric current. However, he did not find any significant difference in the interaction, voltages × days. The encouragement of sprouting by electric current might be due to its stimulation of GA_1_ synthesis as the electric current had an encouraging effect on ZT and GA_1_ in this study and deterring effect on ABA. The electric current channelizes the cellular communication in seed tubers which enhances the translocation of food reserves from parenchyma cells of the internal part to the eyes of the tuber and used by the developing sprout (Mishra, 2015). This is the first-ever report on the effect of electric current on endogenous ZT, GA_1_, and ABA levels of potato tubers.

In potatoes, extended exposure of tubers to temperatures ≤ 2 or ≥30 °C rapidly terminates the dormancy and starts sprouting upon return to moderate temperatures (Wiltshire and Cobb, 1996). In this study, significant differences for dormancy duration and sprout length in experimental genotypes were observed under the effect of cold pre-treatment in comparison with control. The results agree with those of Muthoni et al. (2014), who found that cold pre-treatment of tubers at 2°C shortened the dormancy duration by 2 weeks in long-dormant cultivars. The findings contradict many previous reports that found cold pre-treatment has a non-significant effect on short dormant cultivars (Van Loon, 1987; Van Ittersum and Scholte, 1993). The dormancy breakage through cold pre-treatment might be due to disruption of membrane integrity by low temperature which further leads to electrolyte leakage and subcellular compartmentation. This natural process leads to the use of starch reserves for providing energy for sprout growth.

Previous work related to the effect of cold pre-treatment on endogenous ABA content indicated that initial concentrations of ABA were positively correlated with dormancy duration and negatively correlated with dormancy break (Sonnewald and Sonnewald, 2014). The findings of this study are in agreement with those of Tosetti et al. (2021) that level of ABA is high during dormancy and drops 10–100 times during dormancy break and sprout growth. There is considerable evidence to support the findings related to zeatin increase under the effect of cold pre-treatment. From the findings of Hartmann et al. (2011), it is obvious that the concentrations of cytokinin in tubers stored under different regimes reduce significantly after harvest of the tubers, remain steady during dormancy, but began to increase at dormancy break. The cytokinin concentrations rose faster under high-temperature storage (25°C) compared to low-temperature storage (4°C). Another study supported the current findings where the tubers were stored at 2°C and found that there was a 20- to 50-fold increase in zeatin contents over 6-week storage, coinciding with the natural break of innate dormancy (Bromley, 2009). Similarly, Suttle (1998) reported that an increase in bioactive cytokinin preceded the onset of sprouting in tubers stored under growth permissive conditions and in tubers held in a cold regime (3°C). Cytokinin contents are low in dormant tubers and increased at the commencement of sprouting (Roman et al., 2016). Support for the increase in GA_1_ content in potato tubers comes from the studies of Suttle et al. (2016) and Lizarazo-Peña et al. (2020), who found the levels of endogenous gibberellins were low during dormancy and increase with the start of bud growth. Again during the sprout growth, the concentration of GA_1_ in tubers drops again.

There has been no report found to date about the effect of gamma rays on breakage of potato tuber dormancy, although a lot of its use is published in the past to extend the dormancy period. Gamma rays are the super energetic form of electromagnetic radiation, the most penetrating compared to any other ray (Kovacs and Keresztes, 2002). Irradiation of tubers at higher doses might cause disruption in hormonal balance. However, its use at lower doses did not have a significant effect on dormancy breakage. This study found that response to the highest dose (3.5 kGy) was significant for ABA, ZT, and GA_1_ levels between genotypes and storage periods.

## Conclusion

Understanding the concept of postharvest dormancy may provide an insight into the variety's selection for either their use as seed potatoes or short- to long-term storage for fresh market. The genotypes with wide-ranging dormancy can be selected by the farmers depending on the time gap between the crop planting–harvesting. Indeed, different dormancy-breaking methods (use of PGRs, electric current, cold pre-treatment, and irradiation) might create more flexibility for planting a consecutive crop. Of all the studied methods of potato tubers treatment technologically feasible on large volumes is only the method with pre-cooling at low temperature. The variant with tubers soaking in 60 mgL^−1^ BAP + 20 mgL^−1^ GA_3_ has shown the best results and is technologically expedient only in case of tubers cutting mechanization (peeling damage) before processing. Furthermore, the role of endogenous PGRs during dormancy progression clearly shows that ABA was highest initially and declined with the passage of time. Alternatively, ZT was lowest at the start of dormancy and increased with time. While GA_1_ showed a different response, it increased between week one and week three in the short dormancy genotypes but decreased in the medium- to long-term dormancy genotypes. In further work, the authors recommend studying the field evaluation of different dormancy-breaking methods on emergence, growth, and yield of the potato crop.

## Data availability statement

The raw data supporting the conclusions of this article will be made available by the authors, without undue reservation.

## Author contributions

Conceptualization: MH and MN. Data curation: IA, MK, BA, and MS. Formal analysis: MH, MN, and BA. Funding acquisition: DV, RM, and ME. Investigation: MH, IA, and M. Methodology: MH, RI, and M. Project administration: IA, M, DV, RM, and AA-G. Resources: FA-H and M. Software: RI, MK, and MS. Validation: MN and MK. Visualization: RI, FA-H, ME, and MS. Writing—original draft: MH. Writing—review and editing: MN, IA, BA, DV, RM, MK, AA-G, and RI. All authors have read and agreed to the published version of the manuscript.

## Funding

This study was supported by the National Research Development Projects to finance excellence (PFE)-14/2022-2024 granted by the Romanian Ministry of Research and Innovation and CASEE Fund for Incentives, project No: CASEE fund 2021-2. Further, the authors extend their appreciation to the Researchers Supporting Project number (RSP2022R483), King Saud University, Riyadh, Saudi Arabia, for providing help to publish this research work.

## Conflict of interest

The authors declare that the research was conducted in the absence of any commercial or financial relationships that could be construed as a potential conflict of interest.

## Publisher's note

All claims expressed in this article are solely those of the authors and do not necessarily represent those of their affiliated organizations, or those of the publisher, the editors and the reviewers. Any product that may be evaluated in this article, or claim that may be made by its manufacturer, is not guaranteed or endorsed by the publisher.
