# Supplementary material for: Postharvest dormancy-related changes of endogenous hormones in relation to different dormancy-breaking methods of potato (Solanum tuberosum L.) tubers
Source: Front Plant Sci. 2022 Aug 10;13:945256. doi: 10.3389/fpls.2022.945256 (PMC9399798; doi:10.3389/fpls.2022.945256)
Supplement: Supplementary file 1 [file Data_Sheet_1.docx]

| 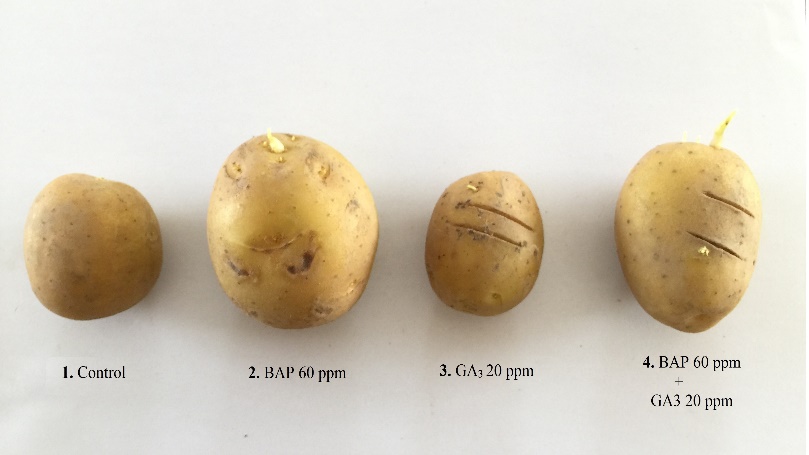 | 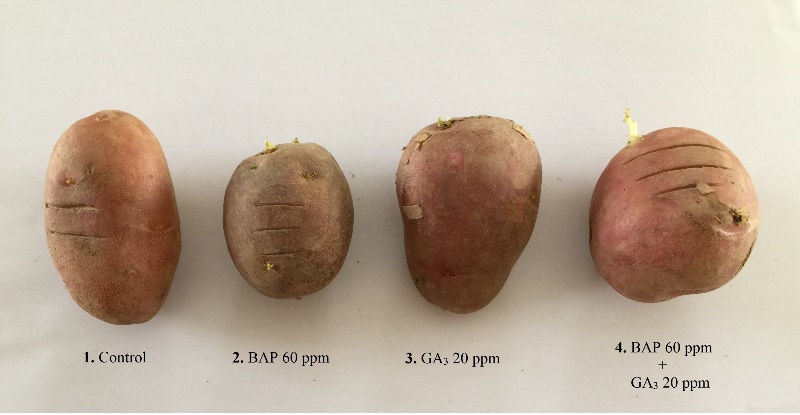 |
| --- | --- |
| **(A)** The sprouting behavior of ‘FD51-5’ in response to BAP and GA_3_ and their combination | **(B)** The sprouting behavior of ‘PRI Red’ in response to BAP and GA_3_ and their combination |
| 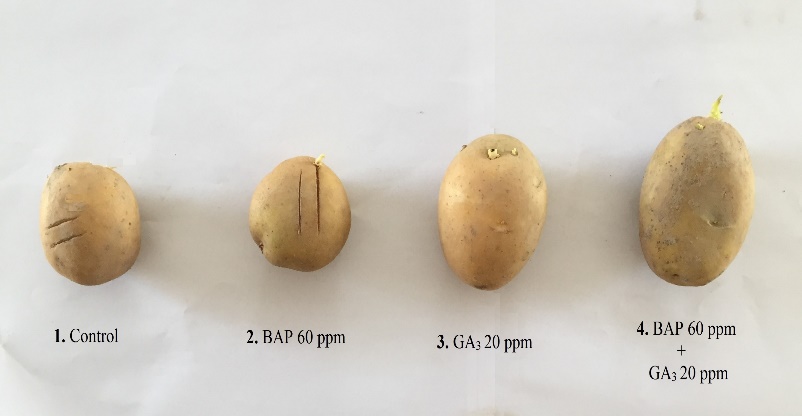 | 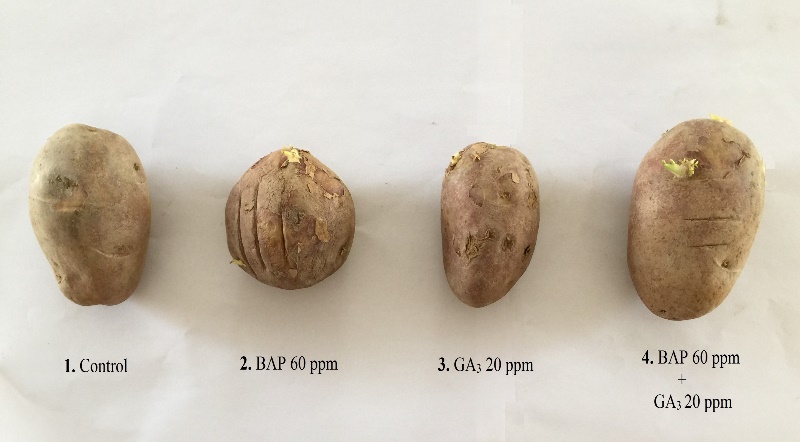 |
| **(C)** The sprouting behavior of ‘Sante’ in response to BAP and GA_3_ and their combination | **(D)** The sprouting behavior of ‘FD73-49’ in response to BAP and GA_3_ and their combination |
| 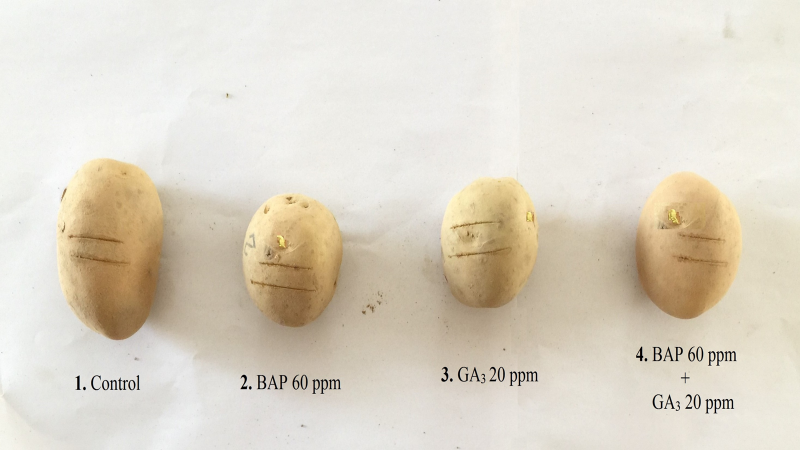 | 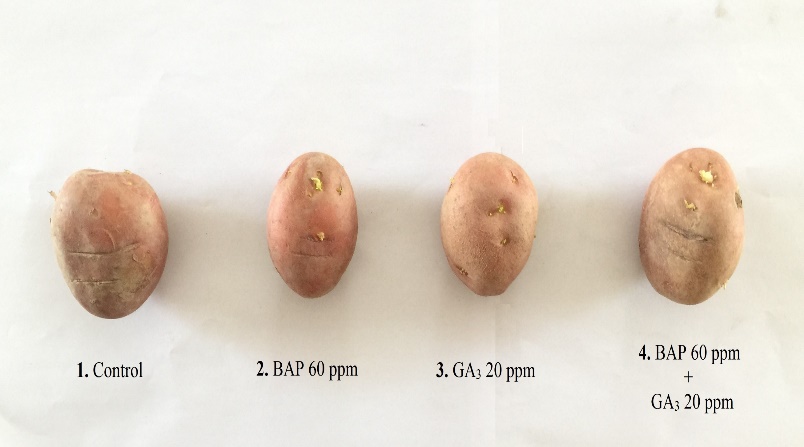 |
| **(E)** The sprouting behavior of ‘FD69-1’ in response to BAP and GA_3_ and their combination | **(F)** The sprouting behavior of ‘FD8-1’ in response to BAP and GA_3_ and their combination |

**Supplementary data**

**Figure 1.** Alone and Combined effect of BAP and GA_3_ on sprouting behavior of six potato genotypes.

| 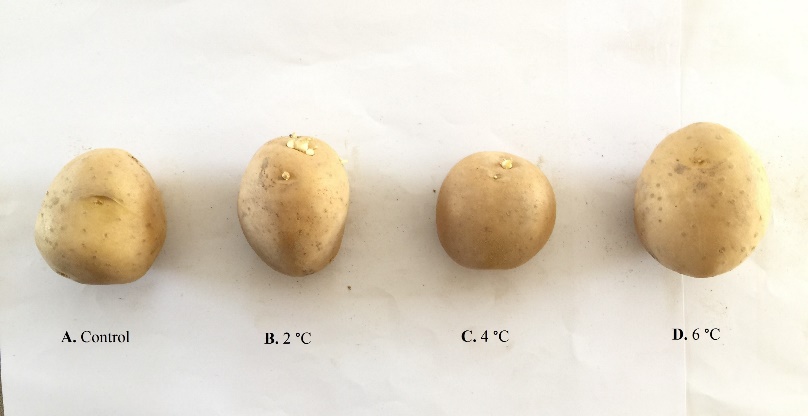 | 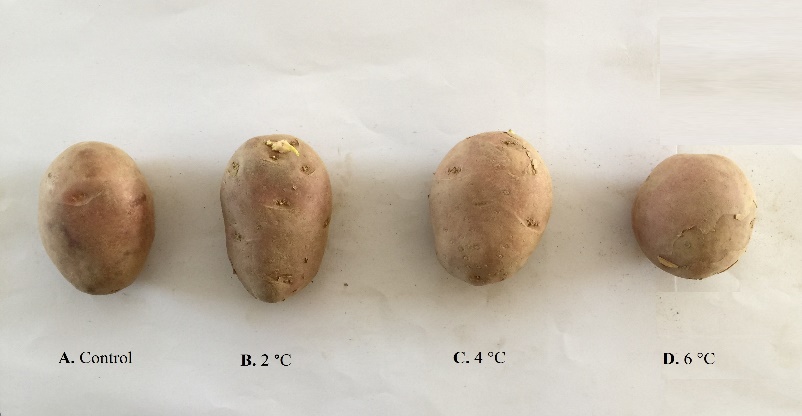 |
| --- | --- |
| **(A)** The sprouting behavior of ‘FD51-5’ in response to low temperature shock | **(B)** The sprouting behavior of ‘PRI Red’ in response to low temperature shock |
| 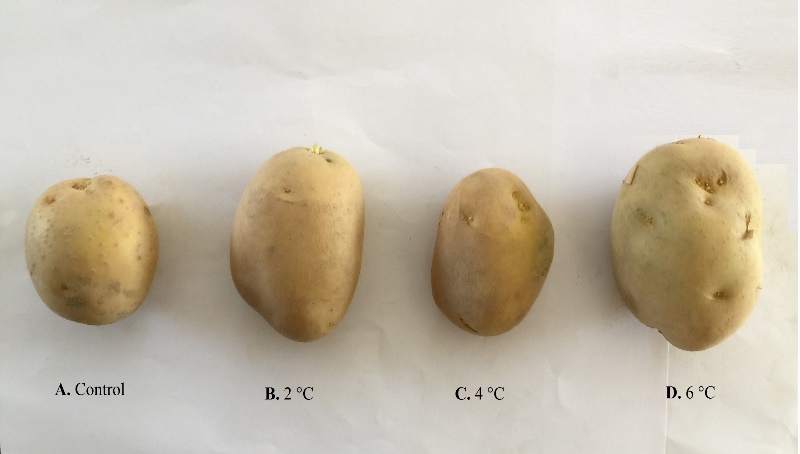 | 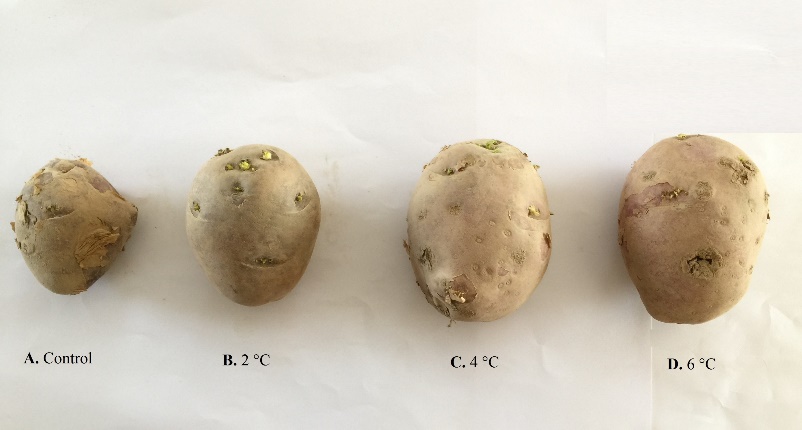 |
| **(C)** The sprouting behavior of ‘Sante’ in response to low temperature shock | **(D)** The sprouting behavior of ‘FD73-49’ in response to low temperature shock |
| 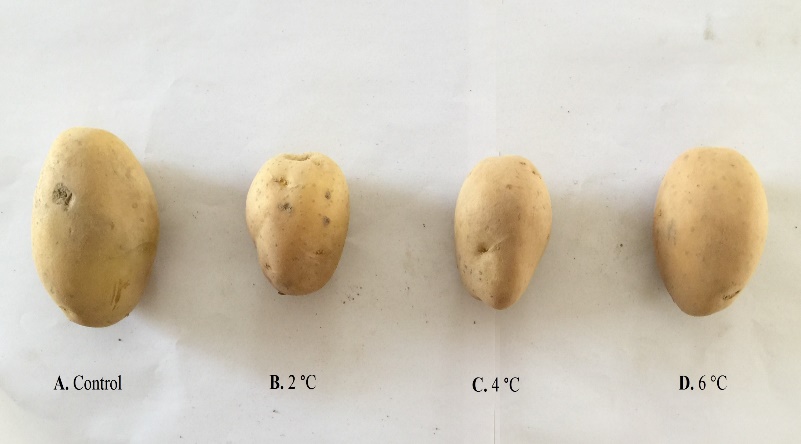 | 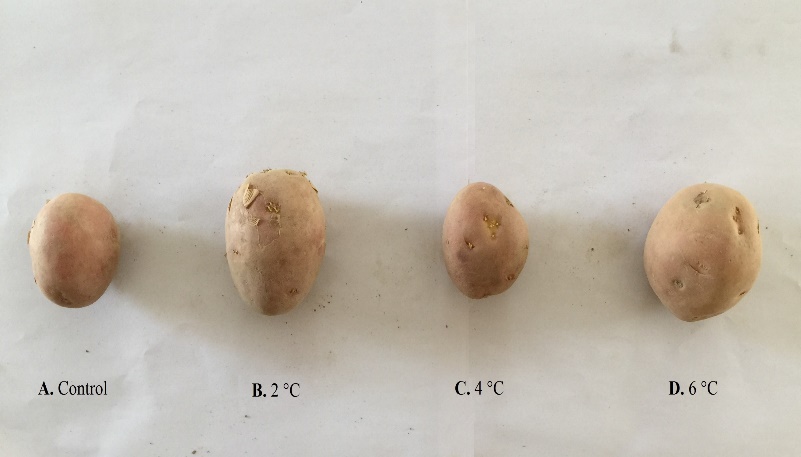 |
| **(E)** The sprouting behavior of ‘FD69-1’ in response to low temperature shock | **(F)** The sprouting behavior of ‘FD8-1’ in response to low temperature shock |

**Figure 2.** Effect of low temperature cold storage on sprouting behaviour of six potato genotypes.

| 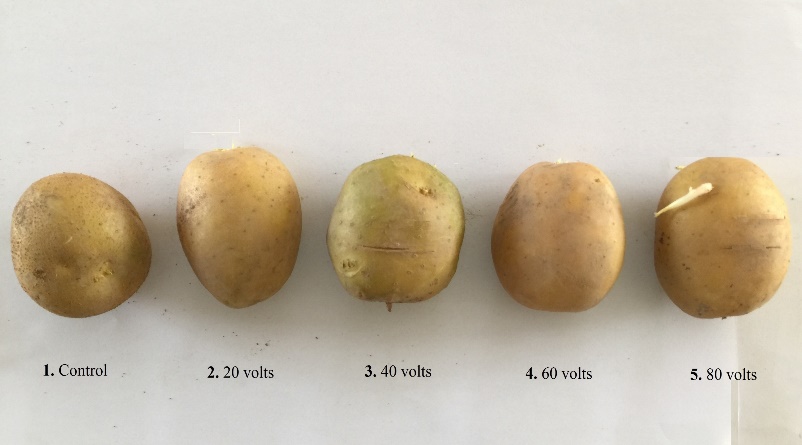 | 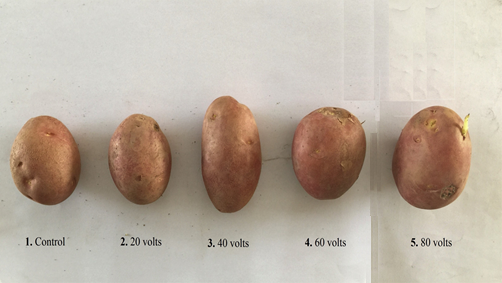 |
| --- | --- |
| **(A)** The sprouting behavior of ‘FD51-5’ in response to BAP and GA_3_ and their combination | **(B)** The sprouting behavior of ‘PRI Red’ in response to BAP and GA_3_ and their combination |
| 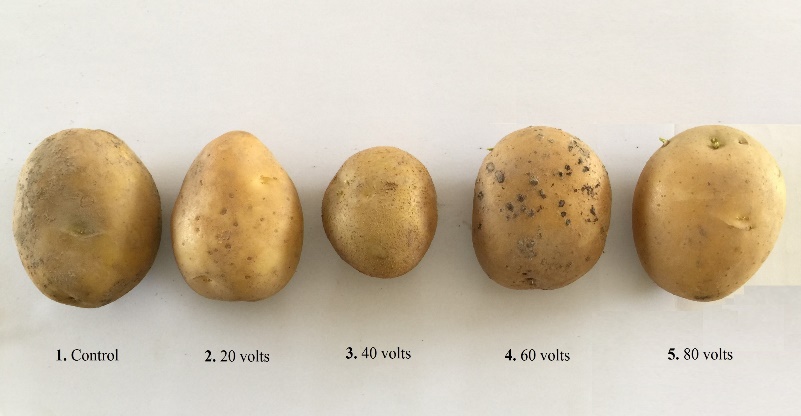 | 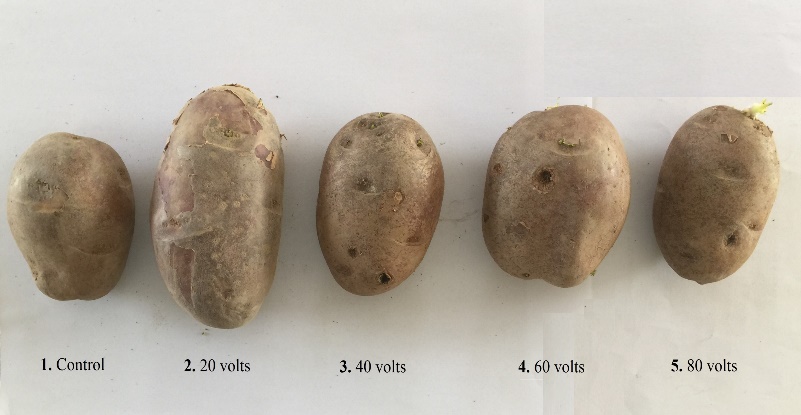 |
| **(C)** The sprouting behavior of ‘Sante’ in response to BAP and GA_3_ and their combination | **(D)** The sprouting behavior of ‘FD73-49’ in response to BAP and GA_3_ and their combination |
| 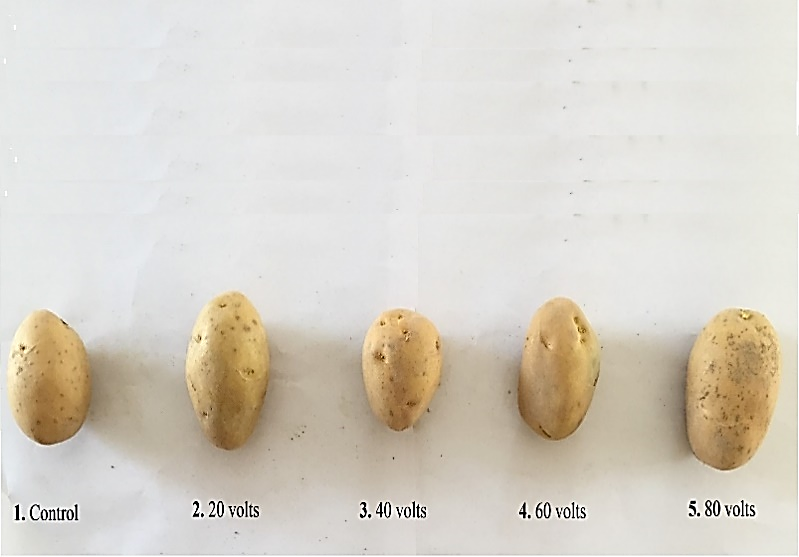 | 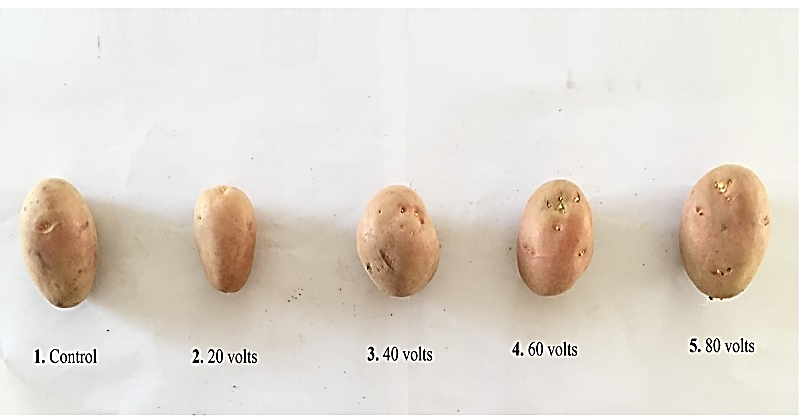 |
| **(E)** The sprouting behavior of ‘FD69-1’ in response to BAP and GA_3_ and their combination | **(F)** The sprouting behavior of ‘FD8-1’ in response to BAP and GA_3_ and their combination |

**Figure 3.** Effect of direct electric current on sprouting behavior of six potato genotypes.

| 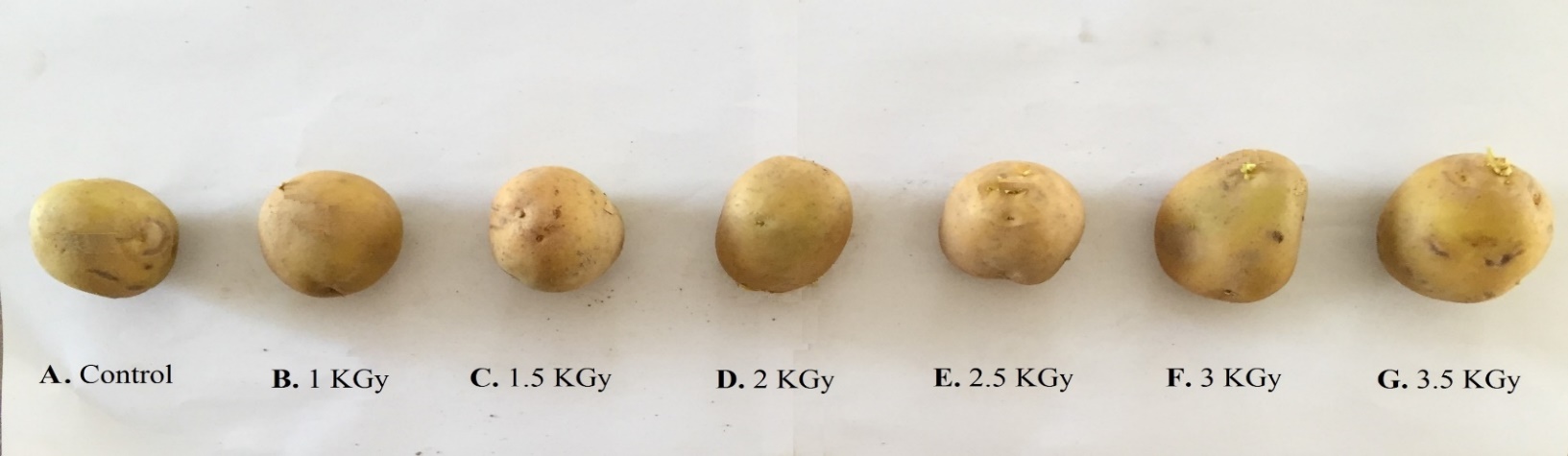 |
| --- |
| **(A)** The sprouting behavior of ‘FD51-5’ in response to irradiation |
| 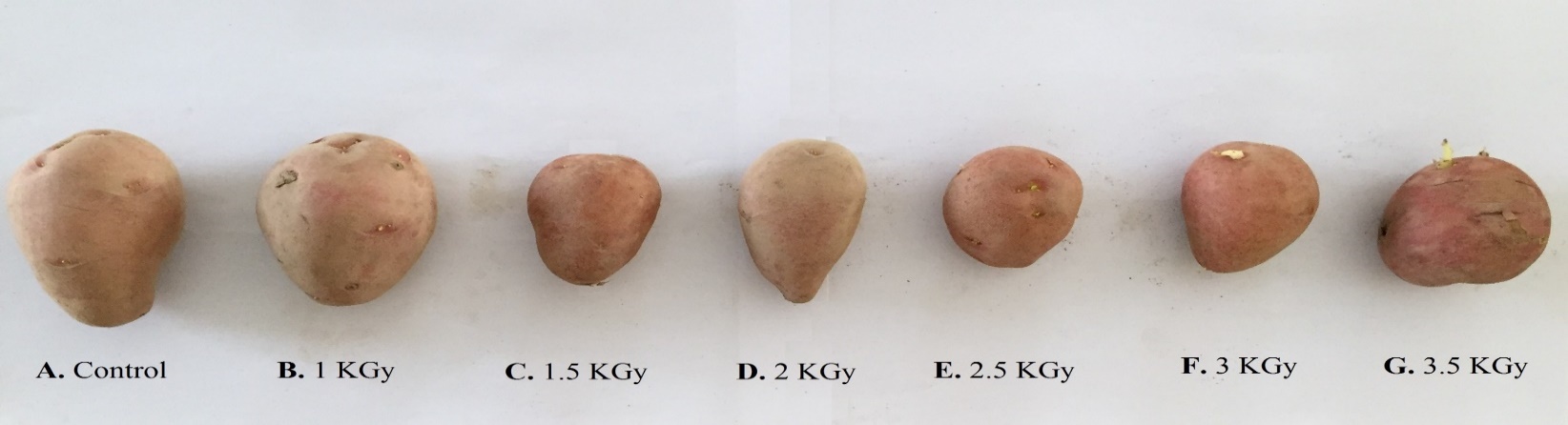 |
| **(B)** The sprouting behavior of ‘PRI Red’ in response to irradiation |
| 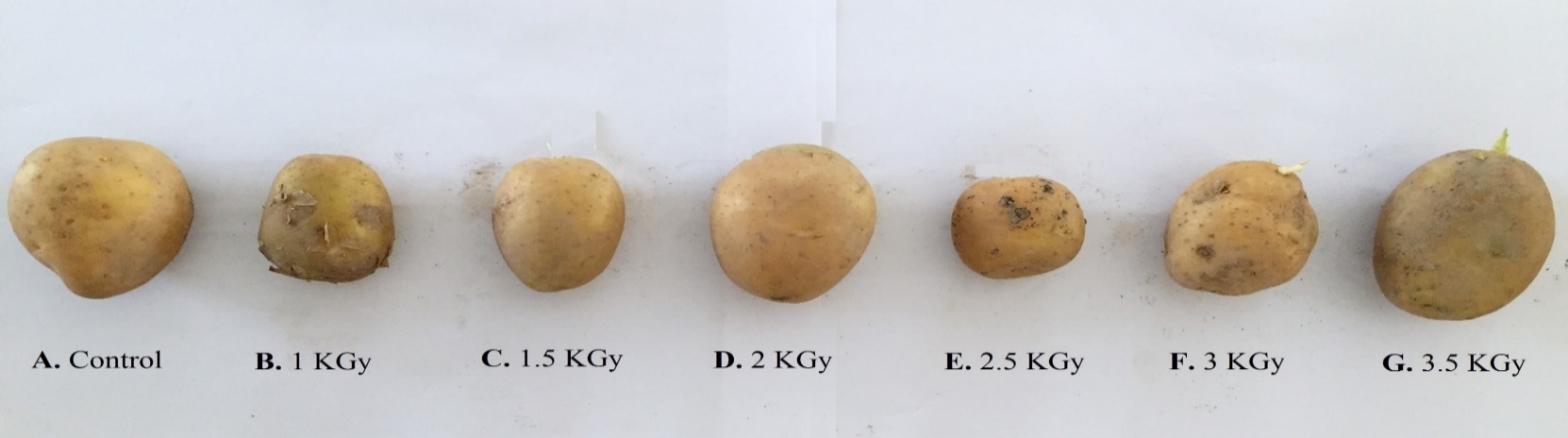 |
| **(C)** The sprouting behavior of ‘Sante’ in response to irradiation |
| 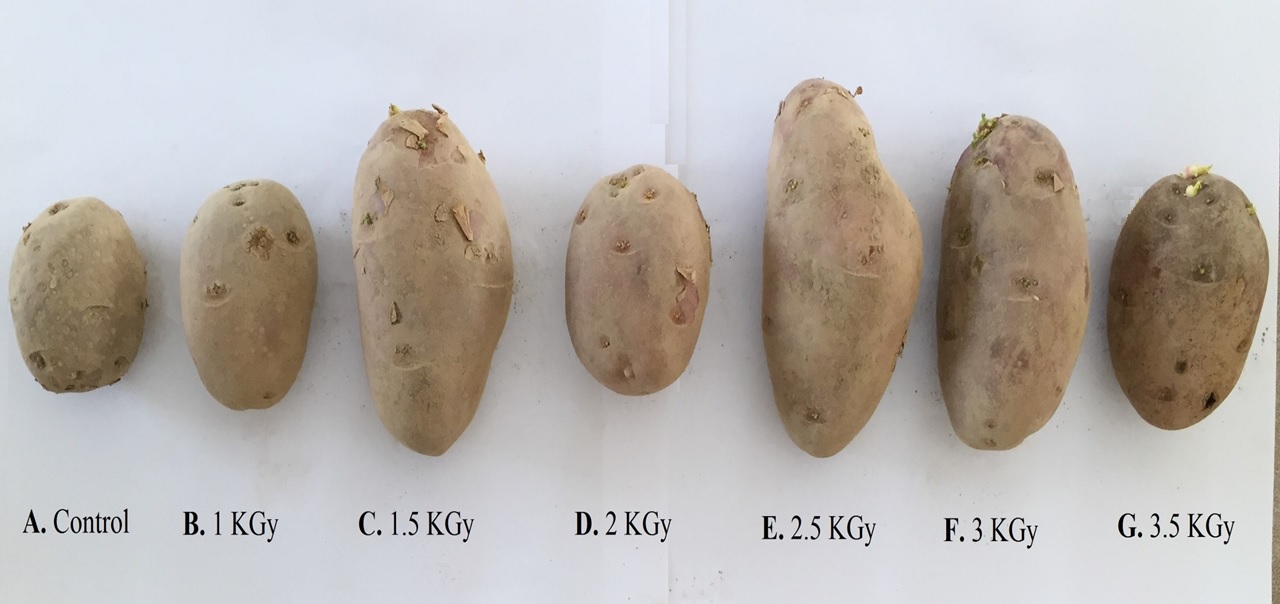 |
| **(D)** The sprouting behavior of ‘FD73-49’ in response to irradiation |
| 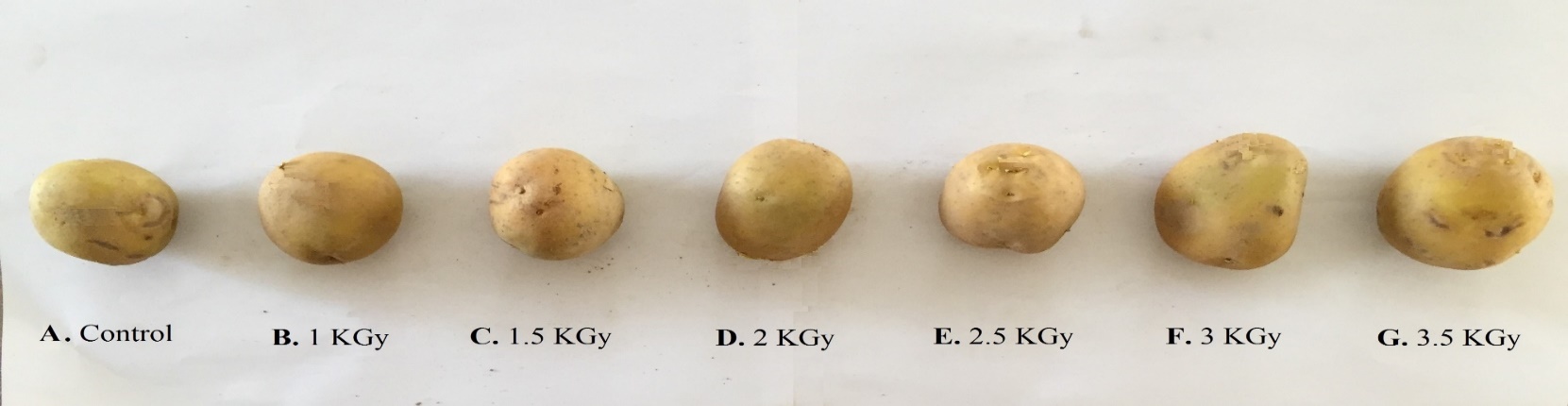 |
| **(E)** The sprouting behavior of ‘FD69-1’ in response to irradiation |
| 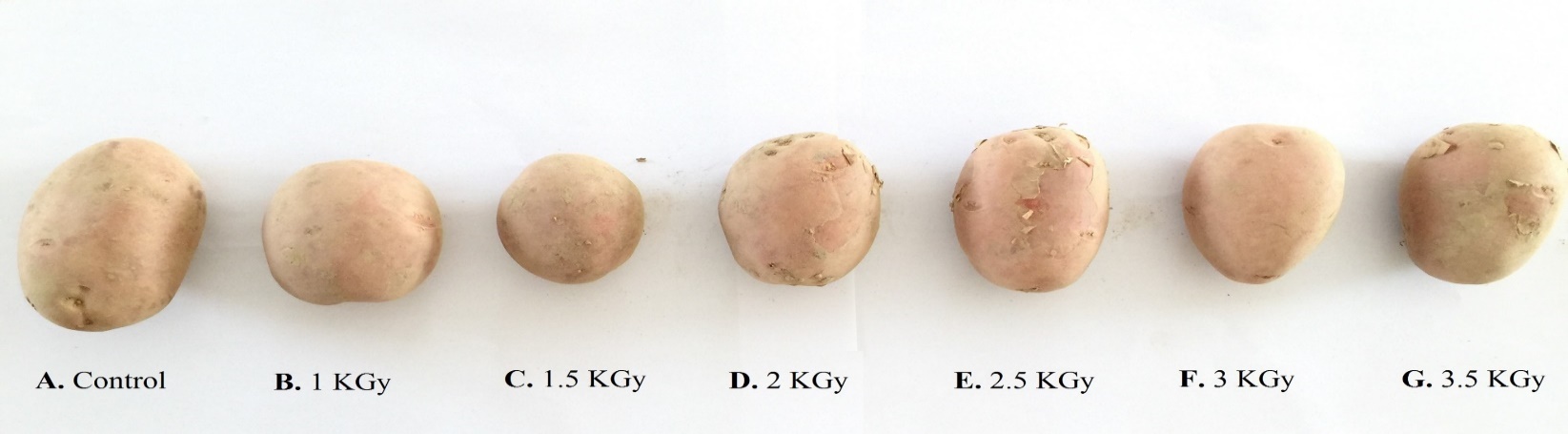 |
| **(F)** The sprouting behavior of ‘FD8-1’ in response to irradiation |

**Figure 4.** Effect of γ-radiations on sprouting behavior of six potato genotypes.
